# Supplementary material for: The Senior Companion Program Plus (SCP Plus): Examining the Preliminary Effectiveness of a Lay Provider Program to Support African American Alzheimer’s Disease and Related Dementias (ADRD) Caregivers
Source: Int J Environ Res Public Health. 2023 Apr 3;20(7):5380. doi: 10.3390/ijerph20075380 (PMC10094539; doi:10.3390/ijerph20075380)
Supplement: Supplementary file 1 [file ijerph-20-05380-s001.zip › ijerph-2284238-supplementary.pdf]

Supplemental Table S1: Characteristics of SCs at Pretest (*N* = 20)

	Total (N = 20)	
Characteristics	Median or n (%)	Range
Age	71.0	58-80
Women	20 (100.0%)	
Marital status		
widowed	8 (40.0%)	
divorced	8 (40.0%	
other	4 (20.0%)	
Education		
high school graduate or below	10 (50.0%)	
some college	4 (20.0%)	
college graduation or above	6 (30.0%)	
Religious service		
at least once a month	5 (25.0%)	
at least once a week	9 (45.0%)	
nearly everyday	6 (30.0%)	
Length of care (years)	4.5	1-15
Daily care (hours)	30.0	6-40
Confidence		
a little bit confident	3 (15.0%)	
confident	10 (50.0%)	
very confident	7 (35.0%)	
Financial Strain		
a little bit difficult	2 (10.0%)	
difficult	10 (50.0%)	
very difficult	8 (40.0%)	
SRH	3.0	1-4
CR's ADLs Needs	2.5	0-6
CR's IADLs Needs	7.5	4-8

Supplemental Table S2: Characteristics of CGs in 3 sites at pretest (*N* = 20)

Variables	Total (N = 20) Median or n (%)	Range
Age	60.5	41-80
Women	17 (85.0%)	
Marital status		
married	11 (55.0%)	
divorced	5 (25.0%)	
widowed/never married/others	4 (20.0%)	
# of people live together	2.0	0-7
Live with CR (yes)	16 (80.0%)	
How long live with CR (years)	3.0	0-48
Live together to take care of CR	15 (75.0%)	
Days others helped last week	1.5	0-7
Education		
high school graduate or below	8 (40.0%)	
some college	5 (25.0%)	
college graduate or above	7 (35.0%)	
Employment (full time)	9 (45.0%)	
Employed outside of home (yes)	15 (75%)	
Hours per week at paid job	30.0	0-60
Reduce hours to take care of CR	7 (35%)	
Stop working because of CR	2 (10.0%)	
Importance of spirituality	3.5	1-4
Religious service attendance	3.0	1-5
How often pray or meditation	5.0	3-5
Relationship to CR		
spouse	1 (5.0%)	
children	13 (65.0%)	
siblings	3 (15.0%)	
others	3 (15.0%)	
Length of care (years)	5.0	1-26
Daily care (hours)	12.0	2-24
Frequency of formal service		
One or less than once a week	1 (5.0%)	
Several times a week	16 (80.0%)	
Everyday	3 (15.0%)	
Satisfaction with formal service		
unsatisfied/strongly unsatisfied	2 (10.0%)	
satisfied/strongly satisfied	18 (90.0%)	

Financial strain		
no or a little bit difficult	4 (20.0%)	
difficult	8 (40.0%)	
very difficult	8 (40.0%)	
SRH of CR	1.5	1-4
SRH of CG	3.0	1-5
Memory of CR	25.0	10-49
